# Phylogeny of multiple genomic regions of infectious laryngotracheitis virus in Turkish poultry flocks

**DOI:** 10.1016/j.psj.2025.104957

**Published:** 2025-02-27

**Authors:** O. Aydin, E. Bayraktar, HE Tali, I.E. Ozkan, A. Yilmaz, S. Umar, OE. Bamac, N. Turan, C. Konuk, Jean-Remy Sadeyen, Pengxiang Chang, JA. Richt, M. Iqbal, H Yilmaz

**Affiliations:** a Department of Virology, Veterinary Faculty, Istanbul University-Cerrahpasa, Buyukcekmece, Hadimkoy, Istanbul, Turkey; b Poultry Division, CEVA Animal Health, Maslak, Turkey; c The Pirbright Institute, Ash Road, Pirbright, Woking, GU24 0NF, UK; d Global Health Research Center (GHRC), Duke Kunshan University, China; e Division of Natural & Applied Sciences (DNAS), Duke Kunshan University, China; f Department of Pathology, Veterinary Faculty, Istanbul University-Cerrahpasa, Buyukcekmece, Hadimkoy, Istanbul, Turkey; g Department of Diagnostic Medicine and Pathobiology, College of Veterinary Medicine, Kansas State University, Manhattan, USA; h Department of Veterinary Tropical Diseases, Faculty of Veterinary Science, University of Pretoria, Onderstepoort 0110, South Africa

**Keywords:** Infectious laryngotracheitis virus, gB gene, gG gene, ICP4 gene, phylogeny

## Abstract

Infectious laryngotracheitis (ILT) is an economically significant respiratory tract viral disease affecting poultry worldwide. There is a scarcity of data on the types of ILTV strains circulating in Turkey. This study aimed to determine the frequency and genotypic variations of Turkish ILTV strains. Commercial layer flocks (*n* = 14) and broiler flocks (*n* = 105) with a history of respiratory diseases were visited. From each flock, 5 to 10 birds from different age groups were necropsied. Clinical and pathological lesions were recorded, and tracheal tissue samples were collected for further studies. Nucleic acid was extracted from samples and subjected to ILTV detection using PCR assays. Clinical signs of anorexia, lethargy, swollen eyelids, mild to severe conjunctivitis, mucoid to purulent nasal discharge, and a drop in egg production were generally observed among ILTV-infected flocks. Pathological lesions, including conjunctivitis, mucoid to purulent sinusitis, and hemorrhagic tracheitis, were observed during necropsy. Among 119 flocks (14 layers and 105 broiler) analyzed in this study, 17 (17/119, 14.28 %) flocks were found positive for ILTV infection by PCR. Of the 17 ILTV-positive samples, 15 could be sequenced successfully for partial gB, gG, and ICP4 genes. Comparative analysis of partial ICP4 gene nucleotides revealed a unique 18 bp insertion "GCGGTTCTTGCGGTTGTT" among ILTV strains. Two nucleotide substitutions were observed in gB gene sequences at positions 5 (T to C) and 488 (A to G), resulting in amino acid substitutions at positions 2 (I to T) and 163 (K to R). Phylogenetic analysis of the gB gene revealed a close clustering (Cluster I) between ILTV strains from this study and those reported from China, Australia, and the USA. Phylogenetic analysis of gG gene sequences showed a close relation to ILTV strains from Russia, China, Canada, the USA, and Italy. No recombination events were observed among the partial sequences of ILTV genes analyzed in this study. Findings of this study show that ILTV infections are frequent in Turkish poultry flocks and contribute to our understanding of the genomic variations in gB, gG and ICP4 genes of ILTV which might help to mitigate ILTV infections in Turkey.

## Introduction

Infectious laryngotracheitis (ILT) is an acute, highly contagious, respiratory viral disease of chickens, pheasants, peafowl, and turkeys, causing economic losses due to reduced egg production, mortality, and cost of control measures (e.g., vaccination, biosecurity) in poultry industries worldwide (Asif et al., 2022; Garcia and Spatz, 2020; Gowthaman et al., 2020; Mo and Mo, 2025). The etiological agent of IL, *Gallid alphaherpesvirus 1*, also known as infectious laryngotracheitis virus (ILTV), is a double-stranded DNA virus, classified in the *Herpesviridae* family, subfamily *Alphaherpesvirinae* and genus *Iltovirus* (Davison, 2010; Garcia and Spatz, 2020). The ILTV genome is about 155 kb linear double stranded DNA consists of 4 distinct regions named unique long (UL), unique short (US), internal repeat (IR) and terminal repeat (TR) region (Gowthaman et al., 2020; Leib et al., 1987). The genome codes about 79 predicted proteins. Envelope glycoproteins (gB, gC, gD, gE, gG, gI, gJ, gK, gL, gM, gN) are embedded in the viral envelope and are crucial for host cell attachment, entry, and cell-to-cell spread (Gowthaman et al., 2020; La et al., 2019; Wild et al., 1996). Nonstructural proteins are not part of the virion but are essential for viral replication, gene expression, and immune evasion. Infected cell protein 4 (ICP4) is an important nonstructural protein which plays a major role in regulation of transcription during virus replication (Fuchs et al., 2002; Ojkic et al., 2006). ILTV field isolates are differentiated from live attenuated vaccine strains based on distinctive differences in nucleotide sequences of the ICP4 region.

Infectious Laryngotracheitis (ILT) was first reported in India in 1964 (Singh et al., 1964) and has since become a global concern, affecting nearly every continent. In Asia, ILTV has been documented in multiple countries, including recent studies from China (Hong et al., 2024; Mo and Mo, 2025; Yi et al., 2024; Zhang et al., 2024), India (Kamal et al., 2024; Priya et al., 2023; Senthilnathan et al., 2024; Tamilmaran et al., 2024), and Bangladesh (Kamal et al., 2024). Similarly, Europe has reported ILTV cases in Switzerland (Hermann et al., 2024) and other regions (Neff et al., 2008; Piccirillo et al., 2016; Zorman Rojs et al., 2021). In Africa, outbreaks have been reported in Egypt (Bayoumi et al., 2020; Ibrahim et al., 2021; Mossad et al., 2022), Ethiopia (Abebe et al., 2024; Mulawa et al., 2024), and other countries (Magouz et al., 2018; Mo and Mo, 2025). The Americas have also faced ILTV challenges, with cases documented in the United States (Blakey et al., 2019; Craig et al., 2017), Canada (Ojkic et al., 2006), and South America (Oldoni and García., 2007; Chacón et al., 2009).

Recent outbreaks in Australia (Agnew-Crumpton et al., 2016; Nazir et al., 2020), Iraq (Alaraji et al., 2019; Ali et al., 2023), and Egypt (Bayoumi et al., 2020; Ibrahim et al., 2021; Mossad et al., 2022) have highlighted the circulation of recombinant strains with increased virulence, posing new challenges for disease control. Additionally, ILTV cases have been reported in Turkey (Can-Sahna et al., 2020; Kardoğan and Sarıçam İnce, 2024; Müştak et al., 2024) and other regions between 2020 and 2024, underscoring its persistent threat. Historically, ILTV outbreaks were documented in over 100 countries between 2000 and 2013 (Menendez et al., 2014), with 88 cases confirmed in California alone during 2007–2017, primarily involving mild clinical forms (Blakey et al., 2019). However, the morbidity and mortality of ILTV outbreaks vary significantly depending on the virulence of circulating strains (Devlin et al., 2006) and the presence of concurrent infections with other respiratory pathogens. The emergence of recombinant strains and the challenges of vaccine efficacy further complicate control efforts, making ILTV a severe and ongoing threat to the global poultry sector. Addressing this threat will require enhanced surveillance, improved vaccines, and global collaboration to mitigate the economic and health impacts of ILTV (Asif et al., 2022; Choi et al., 2016; Craig et al., 2017; Gowthaman et al., 2014; Hermann et al., 2024; Loncoman et al., 2017; Mo and Mo, 2025; Yang et al., 2020; Yilmaz et al., 2004; Zhao et al., 2014; Zorman Rojs et al., 2021). Transmission occurs amongst chicken in the same flock and from farm-to-farm transmission through infected chicken, contaminated dust, litter, beetles, feed, drinking water, vehicles and fomites (Asif et al., 2022; Garcia and Spatz, 2020; Gowthaman et al., 2020; Zorman Rojs et al., 2021). The incubation period is between 6 and 14 days. Mortality, conjunctivitis, nasal discharge, swollen infraorbital sinuses, expectoration of bloody mucus, extension of the neck, dyspnea, gasping, gurgling, rattling, and reduction in weight gain and egg production are the major clinical signs observed in affected flocks (Aras et al., 2018; Garcia and Spatz, 2020; Gowthaman et al., 2020). Chickens recover from infection are long term carriers of the virus as latency occurs after 7 days of acute infection. Intermittent shedding may occur in recovered or vaccinated chickens (Hughes et al., 1991; Williams et al., 1992). Although the vaccination with live attenuated, killed and vectored vaccines (Garcia, 2017) is practiced in infected flocks, once the field virus is established in the farm, it is difficult to control and eradicate since ILTV can survive for 10 days to 3 months at a temperature range of 13-23°C in the environmental conditions particularly in the presence of organic materials (Meulemans and Halen, 1978; Gowthaman et al., 2020).

Field strains and to a certain extend attenuated vaccine strains can be problematic in poultry in terms of diseases occurrence. The mutations and recombination between strains may affect disease severity. Emergence and importance of new virulent strains has been previously reported from different countries (Zhao et al., 2013; Choi et al., 2016; Craig et al., 2017; Yang et al., 2020; Zorman Rojs, 2021; Asif et al., 2022). Therefore, sequence analyses to investigate the variations in the multiple gene regions are crucial to figure out the evolution of ILTV and strain identification which will help to control the epidemics. Fort this, RFLP, partial sequencing, next generation sequencing and whole genome sequencing are commonly used (Ojkic et al., 2006; Craig et al., 2017; La et al., 2019; Asif et al., 2022). For the partial sequencing, TK, UL52, UL54, gB, gC, gB, gJ, ICP1 and ICP4 genes are generally targeted (Choi et al., 2016; Craig et al., 2017; Gowthaman et al., 2014; Zorman Rojs, 2021).

Vaccination in commercial chickens with vector vaccine containing ILTV gene has been going on in Turkey since 2018. There is no comprehensive study on the identification of ILTV strains circulating in Turkish flocks, at present. Because of the importance of the determination of genotypic variations in strains detected in poultry flocks, variations in multiple genomic regions were investigated by partial sequencing of gB, gC and ICP4 genes since they are involved in the early and late stage of ILTV infection. In addition, clinical and pathological observations were also described.

## Materials and methods

### Study design, farms and sampling

This study is based on extensive fieldwork and data collection across multiple regions in Turkey. Commercial poultry farms located in Aegean, Marmara, Inner Anatolia and Western Black Sea regions in Turkey were visited between 2017 and 2022. Commercial layer flocks (*n* = 14) and broiler flocks (*n* = 105) were selected from the regions mentioned above having birds with respiratory symptoms for diagnosis. The flock size of the poultry farms ranged between 10000 and 100000 birds. During the visit observation, biosecurity at the farms was good to moderate. All flocks sampled in this study were not vaccinated against ILTV. Samples were collected from the flocks which had a history of clinical signs compatible with ILT, along with an increase in the mortality rate and decrease in production. 5 to 10 newly died birds, at the age of 56 to 532 days for layers and 20 to 49 days for broilers from each flock, were necropsied and samples from the trachea were taken. The pathological lesions were recorded. The samples were preserved at *−*20°C until processing and analysis.

### DNA extraction

Each trachea sample taken from the chickens was processed individually. About 25 mg of trachea samples were cut into very small pieces and homogenized by using a tissue homogenizer (Next Advance, Bullet Blender, USA, 9A0624H402). DNA was extracted from the homogenized trachea samples using a commercial genomic DNA extraction kit (ThermoFisher Scientific, Invitrogen, Cat No: 182002) as described by the manufacturer. The procedure was completed as performed previously (Yilmaz et al., 2020). The amount of DNA in the extracted material (50 µl) was measured using a NanoDrop spectrophotometer (NanoDrop 1000c, Thermo Scientific, Waltham, USA). Each sample was then adjusted to contain 50 ng DNA for PCR analysis.

### Polymerase chain reaction and sequencing

In order to detect ILTV positive birds, all tracheal samples were analyzed by PCR using the primers for glycoprotein (gB-UL27) gene, PCR reaction and conditions as explained below. To determine genotypic variations in infected-cell polypeptide 4 (ICP4), glycoprotein B (gB-UL27) and glycoprotein G (gG-US4) genes of ILTV, DNA extracts from tracheal tissues were subjected to PCR by using primers specific to gB (Zhao et al., 2013), gG (Gowthaman et al., 2014) and ICP4 (Choi et al., 2016) genes as described previously. For this, an optimized PCR reaction consisted of a total volume of 25 µl reaction mixture containing 12.5 µl Maxima Hot Start PCR Master Mix (Termo Scientific, K1052) and other ingredients including primers, nuclease free water and template DNA as shown in Table 1. The mixture was placed in a thermal cycler (Biorad, Chromo-4), and the PCR conditions are given in Table 1. For all PCR reactions, nuclease-free water was used as negative control in place of template. Positive controls were obtained from samples submitted to the Department of Virology, Veterinary Faculty of Istanbul, and previously confirmed to be ILTV positive by PCR and subsequent sequencing. After amplification, products of gB (567bp), gG (589bp), and ICP4 (603bp) genes seen on agarose gel (1.5%) electrophoresis were sequenced by a commercial company (MedSanTek, Istanbul, Turkey). In case of nucleotide ambiguity, sequencing was repeated. The sequences of gB (PQ520564-PQ520578), gG (PQ520579-PQ520593) and ICP4 (PQ520593- PQ520608) genes from this study were submitted to NCBI GenBank database).

**Table 1:** Primers, reaction mixture and PCR conditions for the target genes, gB, gG and ICP4 for sequencing and phylogenetic analyses.

ILTV Target Genes	Primers (5-3)	Product Size	Reaction Mixture	PCR Conditions	References
**gB (UL27)**	**gB-F:**TTCCGAGATCGAAGAAGTGAG **gB-R:**ACTCTGGTGGCAAGTATCCTGT	567	Mastermix:12,5 µl Primer F: 1 µl Primer R: 1 µl Water: 8,5 µl DNA: 2 µl	95 °C-3 m 94 °C-10 s 60 °C-20 s 72 °C-20 s 72 °C-5 m	Zhao et al., 2013
**gG (US4)**	**p32 U2-F:** CTACGTGCTGGGCTCTAATCC **p32 L2-R:** AAACTCTCGGGTGGCTACTGC	589	Mastermix:12,5 µl Primer F: 1 µl Primer R: 1 µl Water: 8,5 µl DNA: 2 µl	95 °C-3 m 95 °C-1 m 61 °C-1 m 72 °C-1 m 72 °C-1 0m	Gowthaman et al., 2014
**ICP4**	**ICP4-F:** CAAGTTTTTGCCATGGGGAC **ICP4-R:** CATGACAGGCGCAAAAGAC	603	Mastermix:12,5 µl Primer F: 1,5 µl Primer R: 1,5 µl Mgcl_2_: 0,5 µl DMSO: 0,5 µl Water: 4,5 µl DNA: 4 µl	95 °C-3 m 95 °C-30 s 60 °C-45 s 72 °C-1 m 72 °C-5 m	Choi et al., 2016

### Nucleotide and phylogenetic analysis

Partial sequences of gB, gG and ICP4 genes of ILTV were edited and aligned using Clustal-W alignment tool in BioEdit Software (Ibis Biosciences, Carlsbad, CA, USA). Reference sequences for gB, gG and ICP4, genes were retrieved from NCBI GenBank database. Pairwise alignment for respective genes was performed to calculate nucleotide identity between ILTV strains from the present study and reference strains. The evolutionary history was inferred using the Maximum Likelihood method based on the Tamura-Nei model with 1000 bootstrap replicates by using MEGA11 software (http://www.megasoftware.net) (Tamura et al., 2021). The evolutionary distances were computed using the p-distance method. The number of substitutions per site were represented with a scale bar. A total of 36 reference nucleotide sequences were used to generate phylogenetic tree in this study of gB, gG and ICP4 genes.

### Recombination analysis

Recombination analysis and detection of crossover points in the aligned genome sequences were conducted in the Recombination Detection Program (RDP4 v.4.80) (Martin et al., 2015) using default settings.

## Results

### PCR and frequency of ILTV

Out of 14 layers and 105 broiler flocks analyzed in this study, 17 flocks were determined to have ILTV infection by PCR targeting gG gene of ILTV. 15 of the 17 ILTVs were amplified by using the primers targeted to partial gB, gG and ICP4 genes resulting in 567 bp, 589bp and 603 bp product on gel electrophoresis, respectively. Amongst 15 sequenced samples, 1 of them originated from layer flock and the remaining were from broiler flocks.

### Clinical signs

**Fig. 1:**
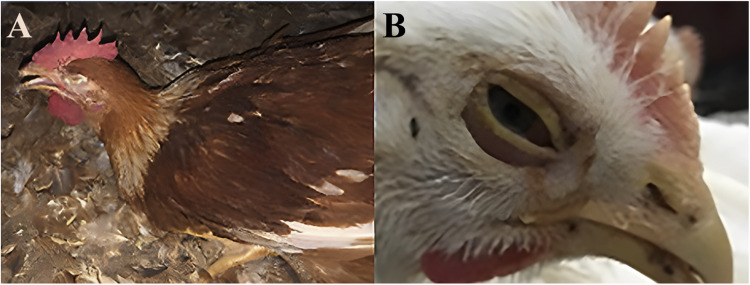
Clinical signs observed in ILTV positive birds: **A:** Extension of the neck due to severe dyspnea; **B:** Severely swollen eyelids, conjunctivitis, ocular and nasal discharges.

Clinical signs of the ILTV positive birds were compatible with both acute and chronic forms of disease. Mortality was about 20% in acutely infected birds. Anorexia, lethargy, swollen eyelids, mild to severe conjunctivitis, mucoid to purulent nasal discharge were seen in most of the cases (Fig. 1**B**). Increased body temperature, dyspnea, gasping with open beak breathing and rattling were the most prominent findings in severely affected birds (Fig. 1**A**). A voice of rattling could be easily heard from the nearby flock. Egg production was also affected in layers. In chronically infected birds, the mortality was much lower (1-2 %) and mild upper respiratory signs were observed characterized by coughing, moist rales, swelling of the infraorbital sinuses (almond-shaped eyes) and drop in egg production up to 30 % and decrease in body weight.

### Gross lesions

**Fig. 2:**
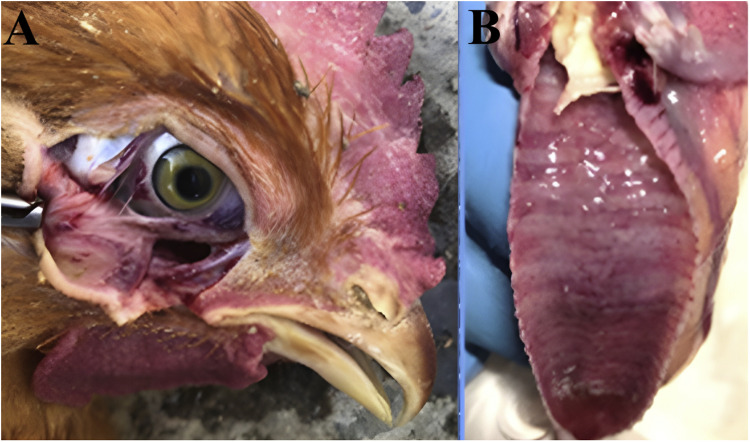
Gross lesions of the ILTV positive birds: **A:** Severe hemorrhagic conjunctivitis; **B:** Fibrino-hemorrhagic tracheitis.

In most birds, gross lesions were restricted to eyes and upper respiratory tract like sinuses, larynx and trachea. Conjunctivitis, swollen eyes and ocular discharge were commonly seen in acutely infected birds (Fig. 2**A**). Mucoid to purulent sinusitis and hemorrhagic tracheitis (Fig. 2**B**) were prominent in most of the cases. Blood cloths were seen in few cases with severe tracheitis. Yellowish caseous diphtheritic membranes in the upper tracheal mucosa and larynx were commonly present. Excessive mucus production was remarkable in the tracheal lumen of the chronically infected birds. Kidneys were swollen and hemorrhagic in some birds. Posterior air sacculates was observed only in one bird.

### Nucleotide and phylogenetic analysis of the and gB, gG and ICP4 genes

**Fig. 3:**
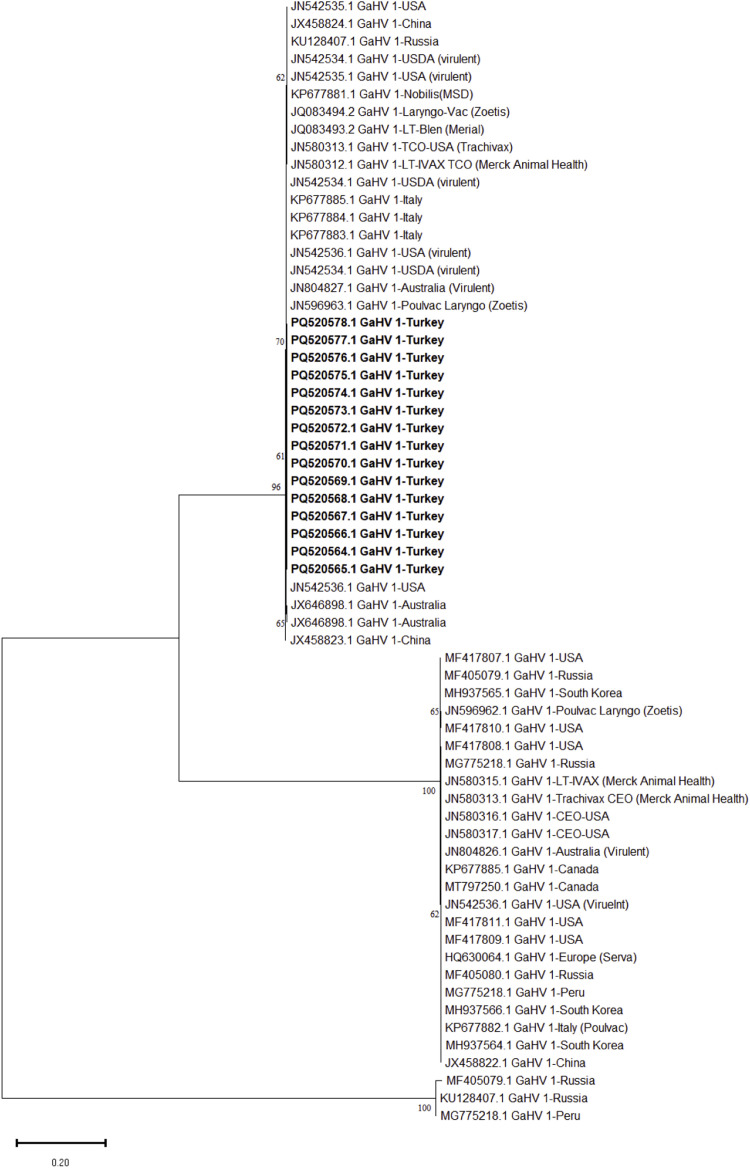
Maximum likelihood tree based on the partial gB gene (US4) sequence of the infectious laryngotracheitis virus (ILTV). ILTV sequences of the present study have been highlighted in “black” triangles”. A total of 36 reference nucleotide sequences of gB gene were used for phylogenetic tree construction in this study. The evolutionary history was inferred using the Maximum Likelihood method based on the Tamura-Nei model with 1000 bootstrap replicates. Bar length indicates the number of substitutions per site.

**Fig. 4:**
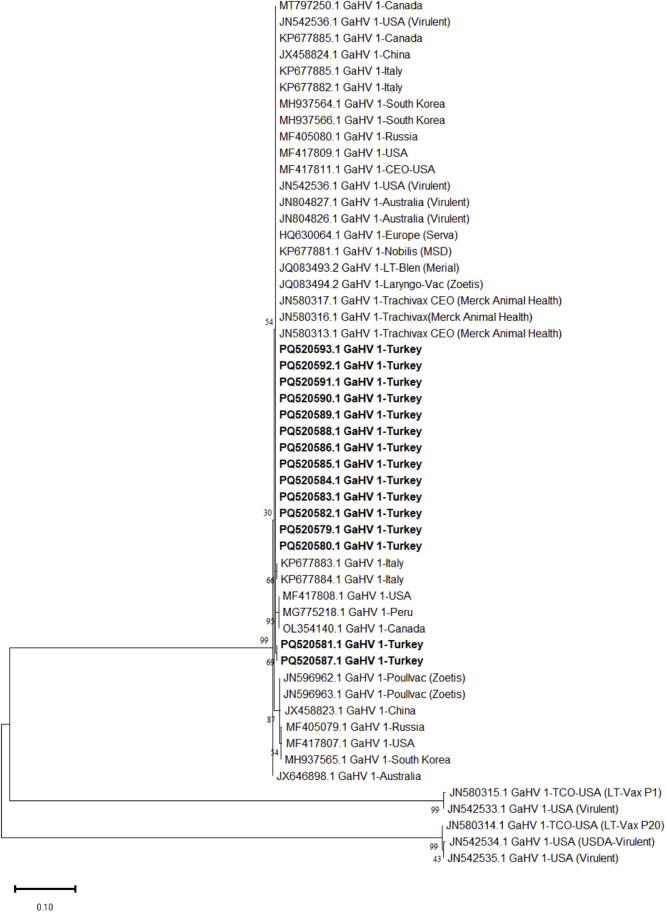
Maximum likelihood tree based on the partial gG gene (UL27) sequence of the infectious laryngotracheitis virus (ILTV). ILTV sequences of the present study have been highlighted in “black” triangles”. A total of 36 reference nucleotide sequences of gG gene were used for phylogenetic tree construction in this study. The evolutionary history was inferred using the Maximum Likelihood method based on the Tamura-Nei model with 1000 bootstrap replicates. Bar length indicates the number of substitutions per site.

**Fig. 5:**
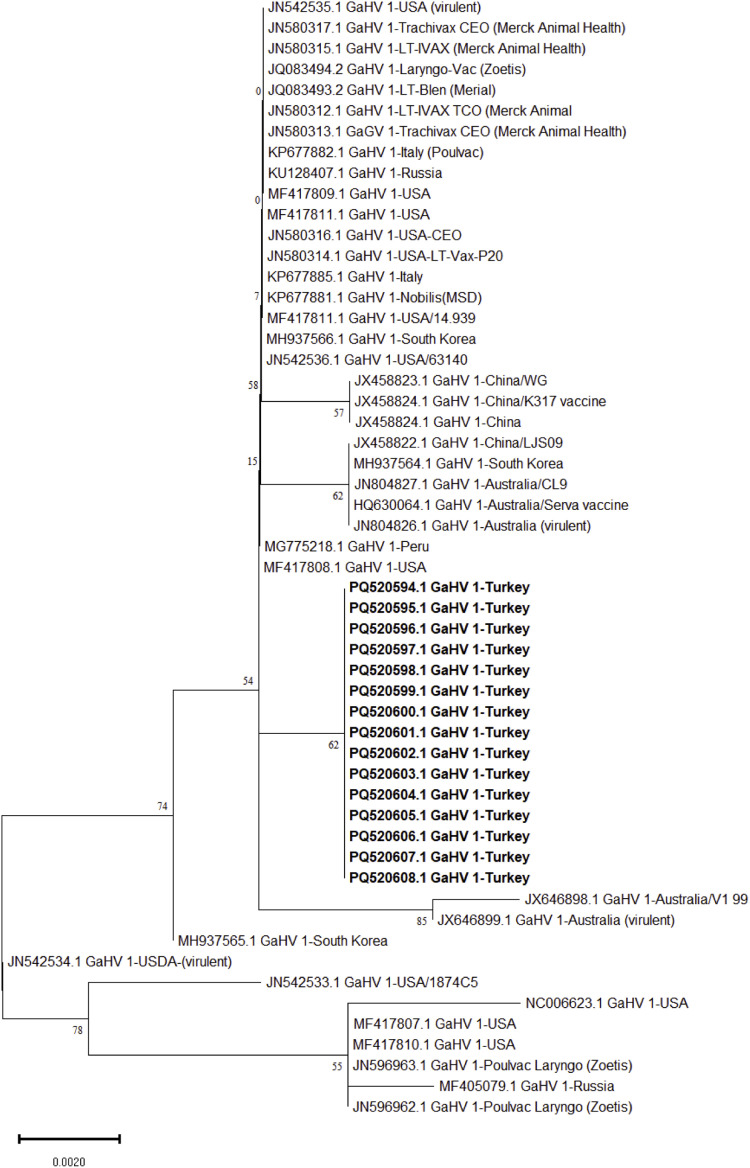
Maximum likelihood tree based on the partial ICP4 gene sequence of the infectious laryngotracheitis virus (ILTV). ILTV sequences of the present study have been highlighted in “black” triangles”. A total of 36 reference nucleotide sequences of ICP4 gene were used for phylogenetic tree construction in this study. The evolutionary history was inferred using the Maximum Likelihood method based on the Tamura-Nei model with 1000 bootstrap replicates. Bar length indicates the number of substitutions per site.

Partial gB, gG and ICP4 genes of 15 ILTVs out of 17 ILTV positive farms could be sequenced successfully. Three phylogenetic trees, based on the partial sequences of gB (567 bp), gG (589 bp) and ICP4 (603 bp) genes of ILTVs were generated (Fig. 3, Fig. 4, Fig. 5). Pairwise comparative analysis showed a nucleotide identity of 99.9-100 % among all partial sequences of gB, gG and ICP4 genes sequences in this study. Comparative analysis of partial ICP4 gene nucleotides revealed a unique 18 bp insertion 131-GCGGTTCTTGCGGTTGTT-148 (genomic nucleotide position 116289 to 116306 with reference to NC-0006623) among all 15 ILTVs strains of the present study. Similar nucleotide insertions were present in ILTV vaccine strains (JN596962, JN596963) and field strains reported previously from US (NC-0006623), Russia (MF405079) and Peru (MG775218). However, this insertion was found absent in reference strains including JN542535 (USA virulent), JN542534 (USDA virulent), JN580312 (LT-Vax), JN580314 (LT-Vax p20), and JN580315 (LT-Vax p1). Beside insertion, nucleotide substitutions were also noted at some positions with reference to NC_0006623 including position 2 (G to A), position 65 (C to T) position 203 (G to A) (**Supplementary Figure 5 and 6**). Deduced amino acid analysis of partial ICP4 protein indicated amino acid substitutions at position 1 (G to E), position 22 (T to I), 68 (G to E), 71 (G to R), 164 (S to L), 191 (V to L). An insertion of six amino acids (corresponding to nucleotide position number 131 to 148) were also observed from position 44 to 49 (CGSCGC) (**Supplementary Figure 5 and 6**).

Two nucleotide substations were observed in gB gene sequences at position number 5 (T to C), and 488 (A to G) which resulted in amino acids substitutions at position 2 (I to T) and 163 (K to R) (**Supplementary Figure 1 and 2**). However, only one nucleotide change was noticed at position 410 of partial gG gene sequences (C to T) which resulted into an amino acid substitution at position 137 (T to M) (**Supplementary Figure 3 and 4**) when compared to reference ILTV strains reported previously (JN580313.1, JN580316.1, JN580317.1, JQ083494.2, JN596962, JN596963)

Phylogenetic analysis based on partial gB gene revealed a close clustering (cluster I) between ILTV strains from the present study and ILTV strains reported form China (JX458823), Australia (JX646898) US (JN542536). They clustered separately (cluster II) with vaccine strains (JN580312, JN580313, JN580314, JN580315, JN580317) and filed strains reported previously Russia (KU128407, MF405079), Peru (MF417808), Canada (KP677885, MT797250) and US (MF417808, MF417807, MF417811) (Fig. 3). A nucleotide similarity of 47.6-99.6% were noticed among ILTV strains from the present study and field and vaccine strains.

Phylogenetic analysis based on the partial gG gene sequences showed that ILTV from the present study were divided into two sub-cluster and were closely associated ILTV strains from Russia, China, Canada, USA and Italy (Fig. 4). Two ILTV strains from the present study (PQ520581, PQ520587) were grouped distantly from all other ILTV strains in the study (Fig. 4). Based on gG gene sequences, a nucleotide similarity of 99.4-99.6 was observed among ILTV strains from the present study. All partial sequences of gG in this study resembled (99.9-100% similarity) with MT797250, KP677885, JN804827 (Fig. 4). There are no sequences of gB and gG genes were reported from Turkey and neighbor countries (Iran, Egypt, Iraq), therefore a direct comparison could not be made in this study.

Phylogenetic analysis based on the ICP4 showed that all ILTV strains obtained in this study clustered separately and were closely related to reference vaccine (JQ083494, JQ083493), and virulent strains from US (MG775216) and Peru (MF417808) (Fig. 5). A nucleotide identity 96-99% were observed between partial ICP4 sequences of ILTV strains in the present study and reference vaccine (JN580312, JN580313, JN580314, JN580315, JN580317, JQ083494, JQ083493, MG775216, MF417808) (Fig. X). However, they were distantly related to other vaccine strains (JN580312, JN580313, JN580314, JN580315, JN580317) and filed strains reported previously from Australia (JX646898, JX646899) and South Korea (MH937565). A nucleotide identity 96-97% were observed among ILTV strains from the present study and previous ILTV vaccine and field strains (JN580312, JN580313, JN580314, JN580315, JN580317, JX646898, JX646899, MH937565). Unfortunately, comparative phylogenetic tree analysis could not be made with ILTVs strains reported from Turkey, Egypt, Iran, Myanmar India and Bangladesh in the past largely due to difference in the region of targeted genes.

### Recombination analysis

All 15 sequences of, gB, gG and ICP4 genes in this study were individually aligned with reference genomes using the MEGA Clustal-W program. Recombination events analysis of ILTV strains sequenced in this study were carried out by using RDP4 software which included six recombination detection tools including RDP, GENECONV, Bootscan, MaxChi, Chimera and SiScan. No recombination events were observed among partial sequences of gB, gG and ICP4 genes in this study.

## Discussion

ILT has been reported in many countries and causing significant economic losses in the poultry industry in Argentina (Craig et al., 2017) Italy (Piccirillo et al., 2016), Australia (Loncoman et al., 2017), South Korea (Choi et al., 206; La et al., 2019), Myanmar (Yang et al., 2020), China, Iraq (Al Saadi et al., 2020) and Turkey (Bayraktar et al., 2019; Kardoğan and Sarıçam İnce, 2024; Mustak and Mustak, 2024) despite the application of preventive measurements. The prevention and control of ILTV is difficult after the initial transmission to flocks and therefore preventive measurements such as good biosecurity and vaccinations are necessary. In addition, it is important to know the circulating strains in chicken flocks for the evolution of viruses that will eventually affect the control of ILT outbreaks, as recombinant viruses outbreaks have been reported recently (Gowthaman et al., 2020; Loncoman et al., 2017;). Therefore, the variations in multiple genes of ILTV, gB, gG and ICP4, were investigated in this study. Previous studies targeted one or two genes to analyze variations in ILTV genes. However, this was not sufficient to find out the changes in the viral genes since ILTV has various genes to be looked. Hence recent studies have investigated allelic variations from multiple target regions of ILTV (Choi et al., 2016).

ILTV is a double-stranded DNA virus with a highly conserved genome. However, mutations and recombination may occur in field and vaccine strains (Zhao et al., 2014). Various genes have been used in order to determine such variations by using sequencing and phylogenetic analyses. In a study reported by Choi and others (2016), 6 different genomic regions including gB and ICP4 were sequenced and results indicated that better discrimination of vaccine and field strains. Their results suggest that the origin of field strain derived from ILTV vaccine could be monitored by using multi-allelic PCR-sequencing method (Choi et al., 2016). In a previous study, sequencing of two different regions of the ICP4 differentiated Brazilian and Peruvian field strains from the vaccine strains indicating that inclusion of ICP4 in the sequencing and phylogenetic studies is important (Chacon and Ferreira, 2009). Therefore, ICP4 was included in the present study to determine variations in the strains circulating in the poultry flocks in Turkey.

Molecular characterization of ILTV is required to distinguish field strains from vaccine strains (Chacon et al., 2009; Choi et al., 2016).ICP4 is an important viral protein involved in the regulation of gene expression in the early stages of infection and is commonly used in epidemiological studies to characterize circulating virus strains (Bayoumi et al., 2020; Shehata et al., 2013). ICP4 has also been considered as a potential differentiation marker due to differences in the ICP4 gene in wild-type and vaccine strains (Chang et al., 1997; Mo and Mo, 2025). Like JN596962, JN596963, MF405079, MG775218, the 18bp insertion was observed in the ILTV strains in this study. This insertion was not present in the TCO and CEO vaccine strains. Based on partial ICP4 gene phylogenetic tree, ILTV strains in this study clustered together with each other, confirming their genetic relatedness. Therefore, It can be assumed that these ILTVs strains in this study may have evolved from a common ancestor. However, they clustered separately with vaccine and wild-type strains of ILTV reported from other countries. Perhaps this is due to the partial ICP4 genome. Notably, the partial gG gene phylogenetic tree demonstrated close clustering of ILTV strains in this study with virulent strains from Italy, USA, Peru, and Canada. This finding suggests a closer genetic relationship with wild-type strains of ILTV. On the other hand, the phylogenetic tree based on the gB gene indicated a close relation with ILTV strains from Australia, USA, and China. None of the ILTV strains in this study clustered with TCO or CEO vaccine strains. Vaccine strains could evolve or recombine with field viruses and thus generating new ILTV strains (Hermann et al., 2024). Comparing ILTV across different countries remains challenging due to the lack of complete genome sequences and a standardized classification method (Hermann et al., 2024). In the USA and Canada, ILTV genotyping primarily relies on the PCR RFLP system introduced by Oldoni and García (2007), which classifies ILTV strains into nine genotypes. While this system has been valuable for regional studies, it has limitations, including its reliance on partial genome sequences and the potential for misclassification due to recombination or convergent evolution. Additionally, the lack of harmonization between genotyping systems used in different regions complicates global comparisons and hinders our understanding of ILTV epidemiology.

Sequence analyses of the ICP4 gene showed that the ILTVs strains in this study were similar ILTV strains from previously reported from Italy, USA, Peru, China and Canada by a range of 98.4 % to 99.8 %. All these findings suggest that these ILTVs strains in the present study may have originated from field strains (Garcia et al., 2019; Menendez et al., 2014). In contrast, the CEO and TCO vaccine strains (JN580312.1 and JN580313) showed only 96.6% identity with the ILTVs strains in this study. It is worth noting that all inferences made in this study should be treated with caution, as these analyses were based on a very small region of the ICP4 gene. This limitation emphasizes the need to further evaluate the whole genome sequences of Turkish ILTV isolates in order to better understand their relationship with the reference strains. Some non-synonymous amino acid substitutions were observed in the ICP4 protein sequence, which may affect the virulence of ILTV (Mossad et al., 2022; Veits et al., 2003). Surprisingly, recombination events were not observed in ILTVs strains in this study. It has been suggested that outbreaks of ILTVs occur after vaccination programs have been developed (Oldoni and García., 2007). Vaccine viruses gradually replace wild strains in the wild (Chang et al., 1997) and their virulence increases during bird-to-bird transmission (Abdel-Moneim et al., 2014; Bayoumi et al., 2020; Shehata et al., 2013). Recombination of vaccine strains and the consequent emergence of new virulent wild isolates has been documented by many researchers (Agnew-Crumpton et al., 2016; La et al., 2019; Lee et al., 2012, 2013). Non-recombinant ILTVs strains are likely to have become endemic in Turkey, leading to disease outbreaks in broilers and laying hens in 2018 and 2022.

Glycoprotein B encoded by the UL27 gene is one of the major proteins of ILTV and plays an important role in viral attachment to target cells and cell entry (Connolly et al., 2011). In this study, a unique point mutation (Lysine (K) to Arginine (R) at position 163) was found in all ILTV strains, but not in the CEO and TCO vaccines. This point mutation, as well as other point mutations in the gB gene sequence, can serve as good discriminatory markers between field and vaccine strains (Garcia et al., 2013; Piccirillo et al., 2016; Yang et al., 2020).

Sequencing analysis of the gG gene has also been used to characterize ILTV isolates (Han et al., 2001). In this study, a unique point mutation was observed in two sequences of ILTV (PQ520581 and PQ520588). Threonine (T) was replaced by methionine (M) at position 137, which has not been documented in previous field and vaccine strains. In plant genomics, the ILTVs strains in this study are very close to the Italian, American, Canadian and CEO vaccine strains.

Clinical signs and necropsy findings observed in this study were similar to those reported in previous studies (Blakey et al., 2019; Gowthaman et al., 2020; Tsiouris et al., 2021; Zorman Rojs et al., 2021). Some reports show that adult birds show severe signs of disease (Gowthaman et al., 2020). However, in a study performed previously, both young and adult birds showed severe respiratory disease that indicates ILTV have similar virulence for all ages (Gowthaman et al., 2014). In addition, results of a study confirmed that there is a considerable variation among ILTV strains in terms of their tropism, clinical signs and lesions in different tissues and capacity to induce mortality (Gowthaman et al., 2014; Kirkpatrick et al., 2006). The characteristic clinical signs and necropsy findings observed in this study correlates with the PCR results that indicates PCR is a useful tool to confirm the presence of ILTV in clinical samples especially when the isolation becomes difficult when other respiratory pathogens exist in diagnostic samples.

## Conclusions

The findings of this study demonstrate that ILTV infections are prevalent in Turkish poultry flocks, highlighting the need for effective control measures. Our analysis of genomic variations in the gB, gG, and ICP4 genes of ILTV provides valuable insights into the virus's genetic diversity, which may influence its virulence, immune evasion, and vaccine efficacy. These findings contribute to a deeper understanding of ILTV epidemiology in Turkey and offer a foundation for developing targeted interventions. Given the persistent threat of ILTV and the emergence of recombinant strains globally, a rigorous nationwide investigation of the genotypic and evolutionary characteristics of ILTV is warranted. Such efforts should focus on differentiating between wild-type and vaccine strains, identifying emerging variants, and understanding their impact on disease dynamics. This will not only enhance local control strategies but also contribute to global efforts to mitigate ILTV infections. By integrating genomic surveillance with improved vaccines and biosecurity measures, Turkey can take a proactive role in reducing the economic and health impacts of ILTV on its poultry industry.

## Declaration of competing interest

The authors declare the following financial interests/personal relationships which may be considered as potential competing interests:

Huseyin Yilmaz reports financial support, administrative support, and equipment, drugs, or supplies were provided by Istanbul University Cerrahpasa. Juergen Richt reports administrative support, article publishing charges, and writing assistance were provided by Kansas State University. Huseyin Yilmaz reports a relationship with Istanbul University Cerrahpasa that includes: employment. None If there are other authors, they declare that they have no known competing financial interests or personal relationships that could have appeared to influence the work reported in this paper.

## Appendix Supplementary materials

Image, application 1

Image, application 2

Image, application 3

Image, application 4

Image, application 5

Image, application 6

Image, application 7
