# Supplementary figures and images for: Phylogeny of multiple genomic regions of infectious laryngotracheitis virus in Turkish poultry flocks
Source: Poult Sci. 2025 Feb 27;104(5):104957. doi: 10.1016/j.psj.2025.104957 (PMC11960647; doi:10.1016/j.psj.2025.104957)

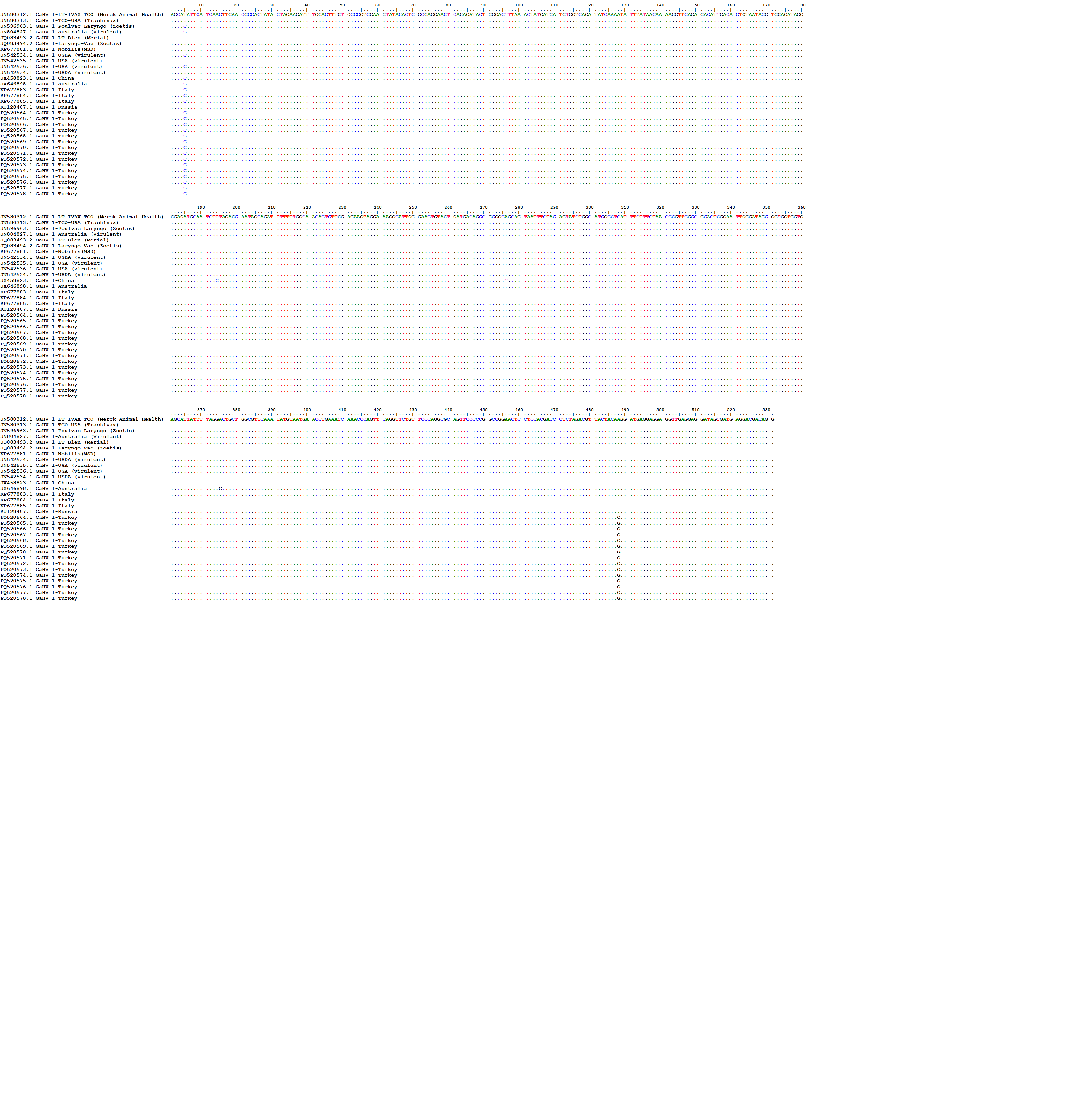

Supplement: Supplementary file 1 [file mmc1.docx]

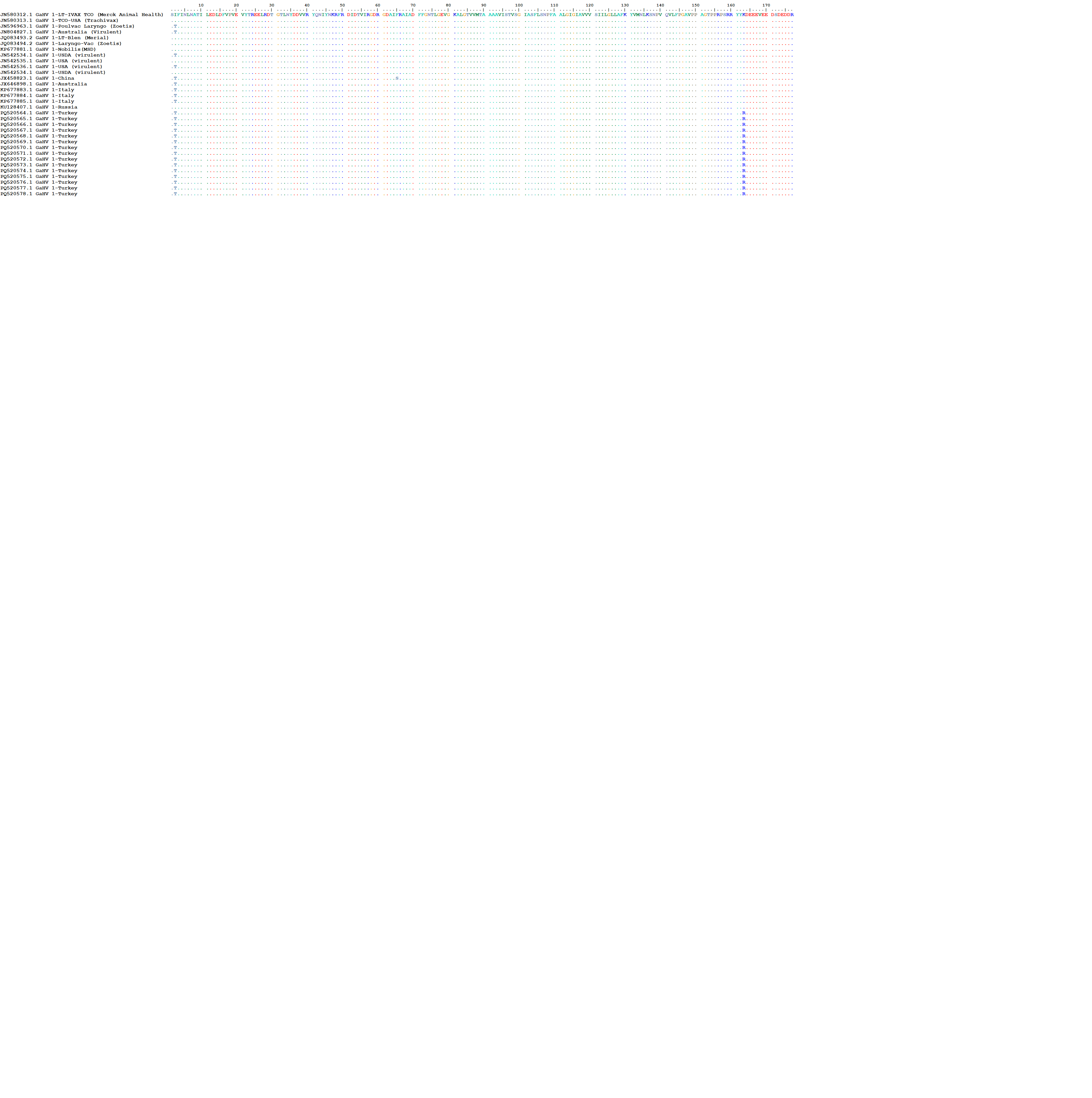

Supplement: Supplementary file 2 [file mmc2.docx]

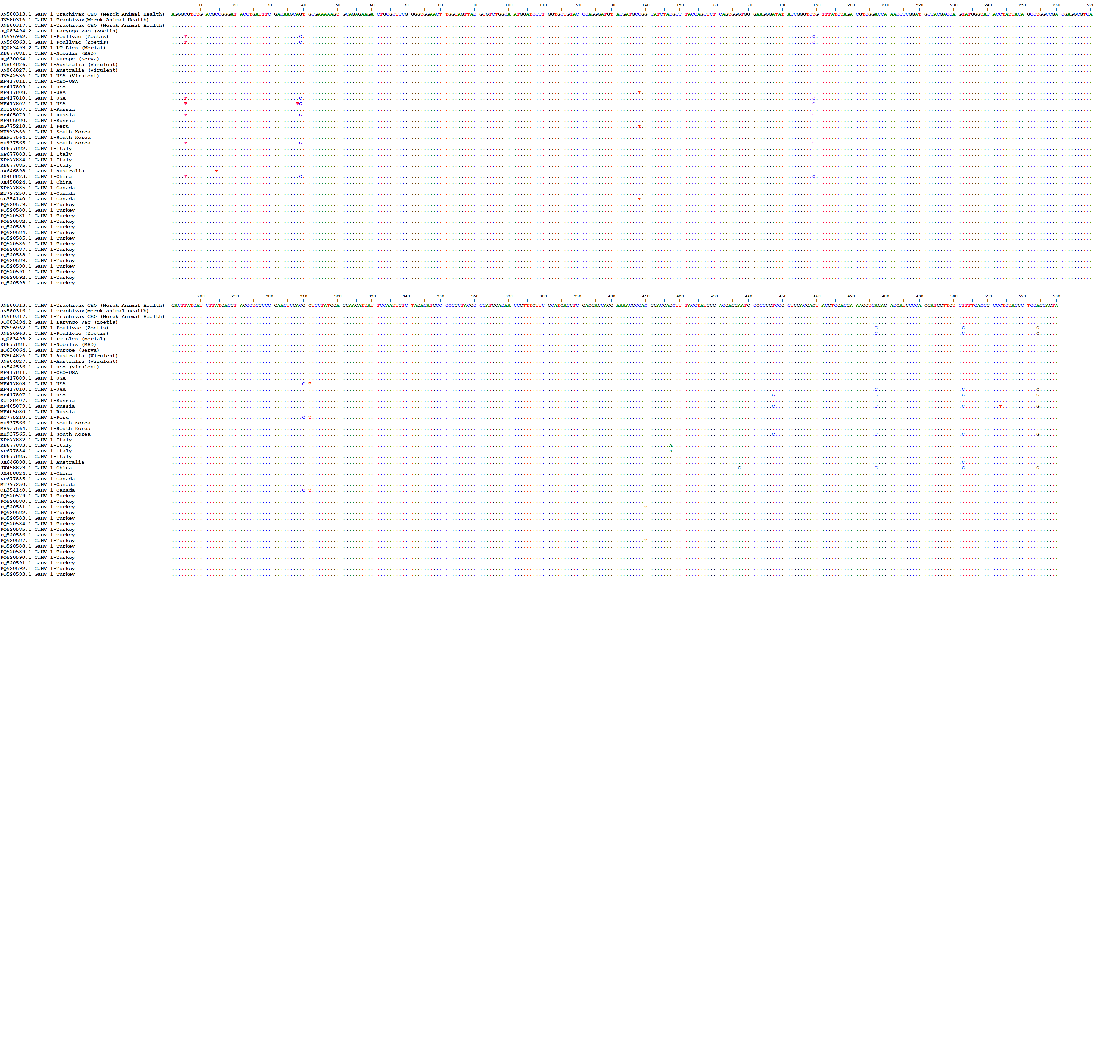

Supplement: Supplementary file 3 [file mmc3.docx]

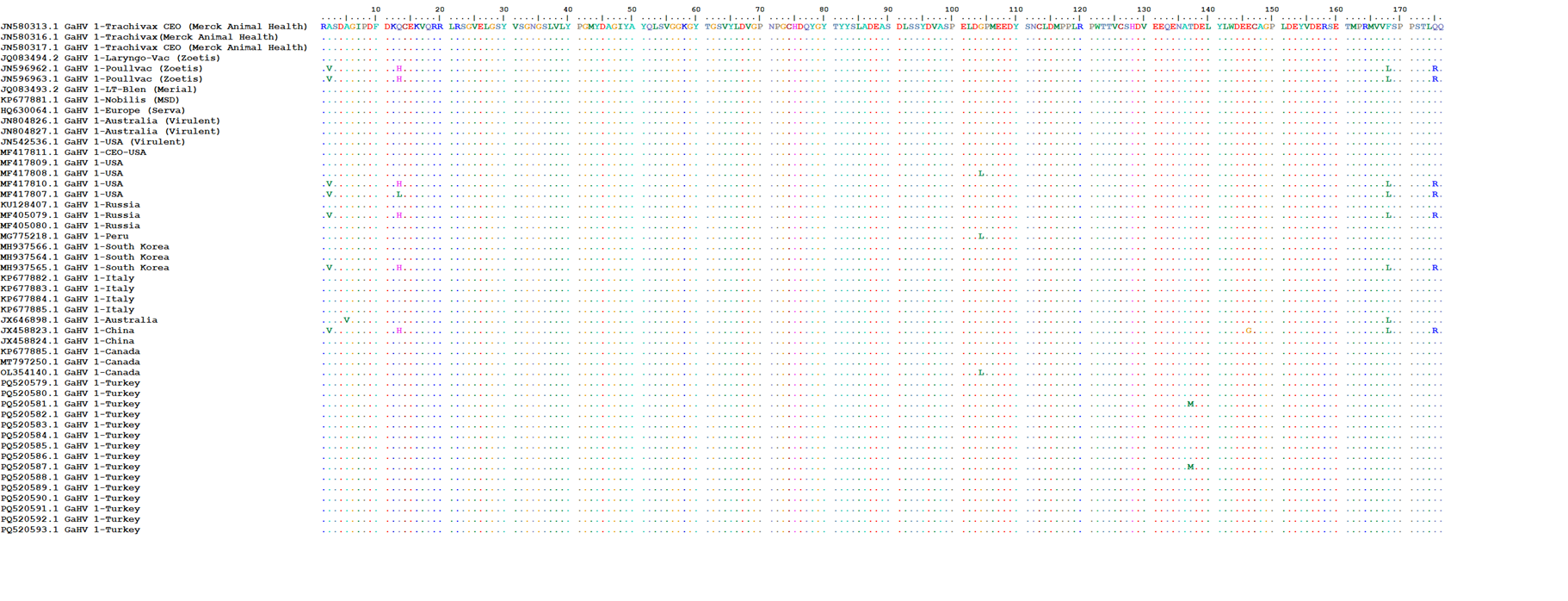

Supplement: Supplementary file 4 [file mmc4.docx]

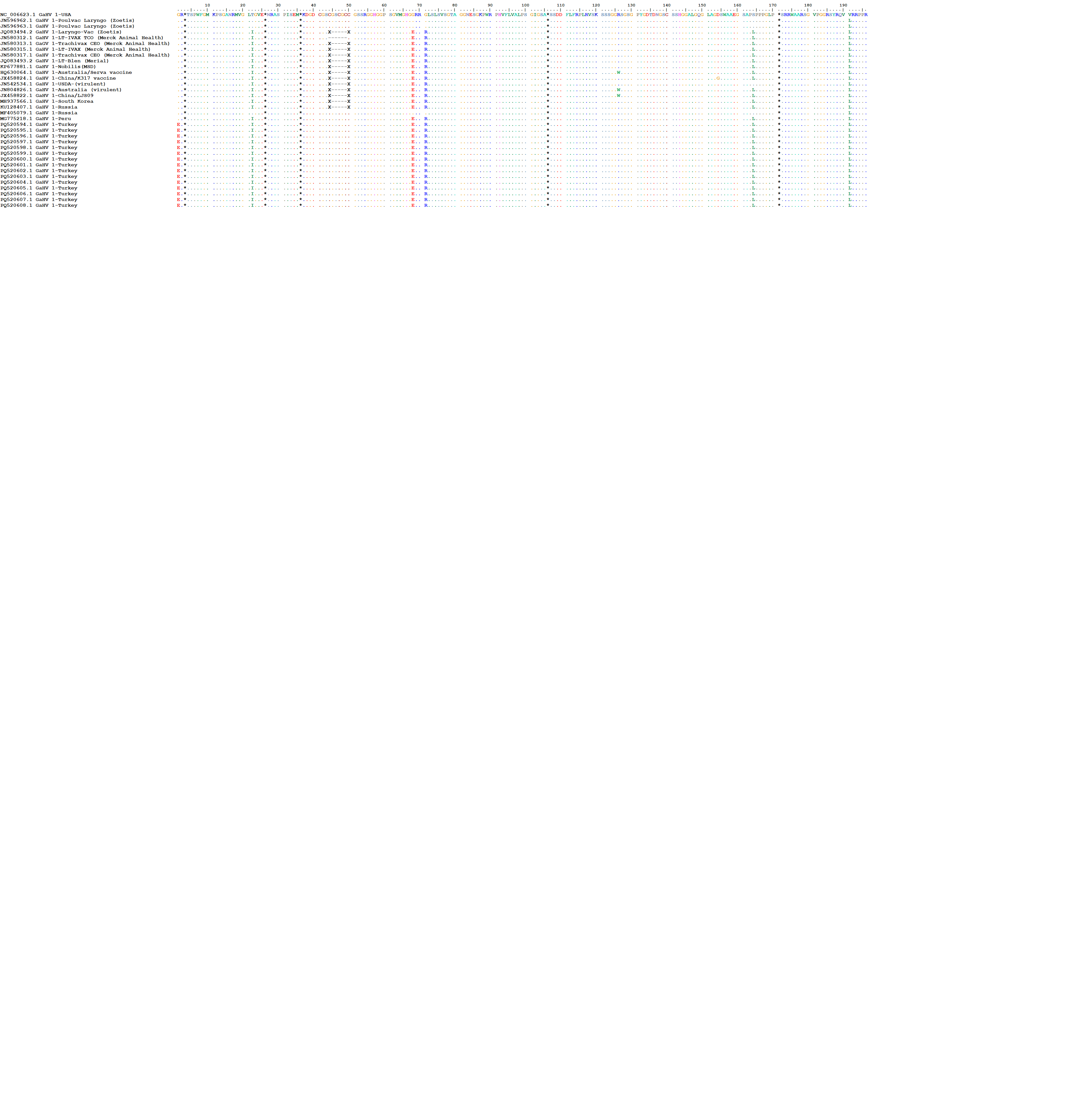

Supplement: Supplementary file 5 [file mmc5.docx]

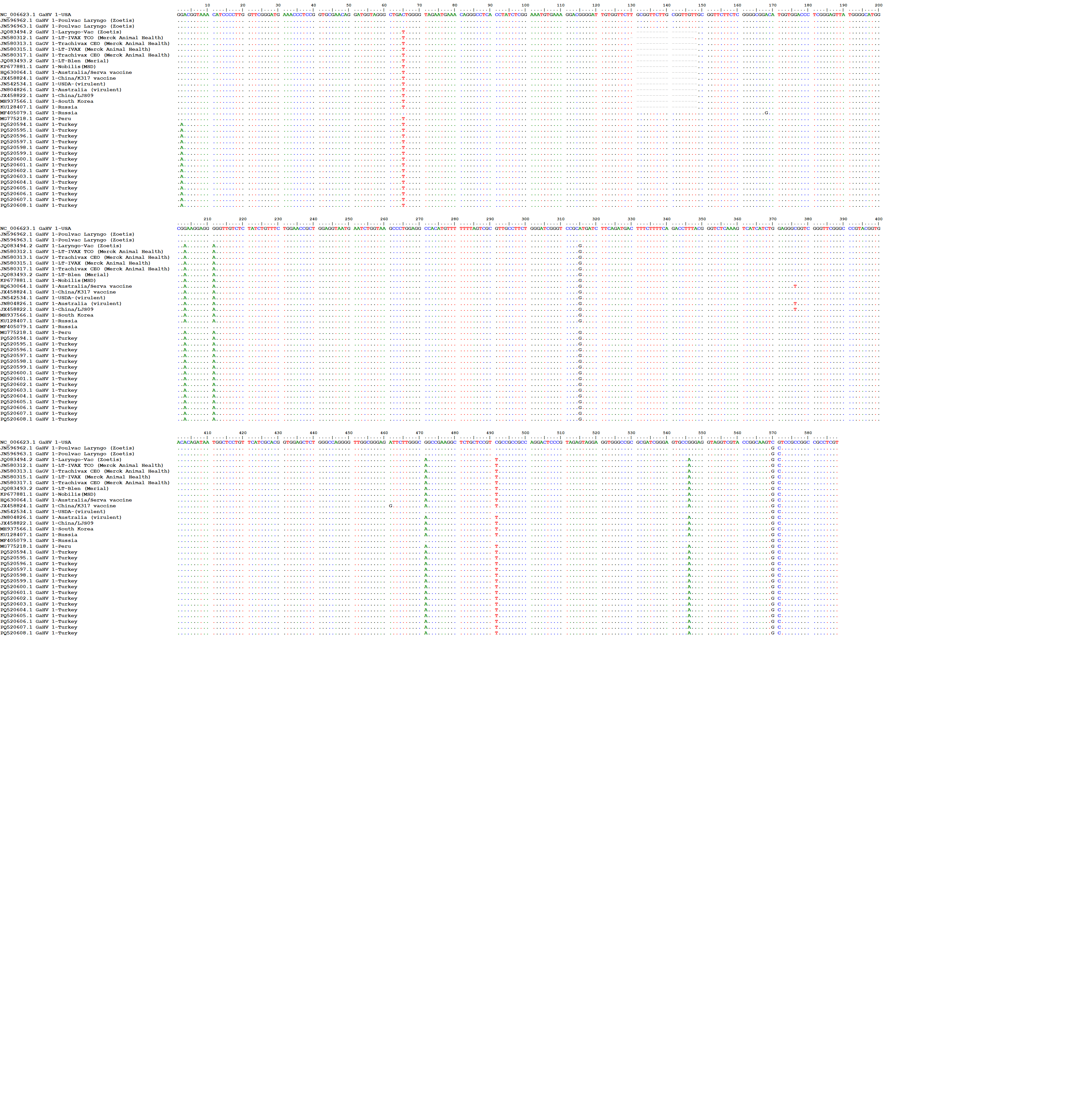

Supplement: Supplementary file 6 [file mmc6.docx]
