# Supplementary material for: Phylogeny of multiple genomic regions of infectious laryngotracheitis virus in Turkish poultry flocks
Source: Poult Sci. 2025 Feb 27;104(5):104957. doi: 10.1016/j.psj.2025.104957 (PMC11960647; doi:10.1016/j.psj.2025.104957)
Supplement: Supplementary file 7 [file mmc7.docx]

**Table S1.** Reference strains of ILTV that were retrieved from GenBank database and used in phylogenetic analysis in this study

| **Strain** | **Vaccine/virulent** | **Country** | **Genome length** | **Accession Number** |
| --- | --- | --- | --- | --- |
| TCO LOW | Vaccine | USA | 155,465 | JN580315 |
| TCO IVAX | Vaccine | USA | 155,465 | JN580312 |
| S2816 | Virulent | USA | 154,001 | MF417807 |
| J2 | Virulent | USA | 153,711 | MF417808 |
| USDA REF | Virulent | USA | 151,756 | JN542534 |
| CEO TRVX | Vaccine | USA | 153,647 | JN580313 |
| 6.48.88 | Virulent | USA | 154,022 | MF417810 |
| 81658 | Virulent | USA | 150,335 | JN542535 |
| 63140/C/08/BR | Virulent | USA | 153,633 | JN542536 |
| 1874C5 | Virulent | USA | 149,682 | JN542533 |
| 3.26.90 | Virulent | USA | 153,655 | MF417809 |
| 14.939 | Virulent | USA | 153,629 | MF417811 |
| SERVA | Vaccine | Europe | 152,630 | HQ630064 |
| O | Virulent | Russia | 153,634 | KU128407 |
| CK/TATARSTAN/2009/1643 | Virulent | Russia | 153,633 | MF405079 |
| 2013/2701 | Virulent | Russia | 153,634 | MF405080 |
| VFAR043 | Virulent | Russia | 153,634 | MG775218 |
| NOBILIS LARYNGOVAC | Vaccine | USA | 152,701 | KP677881 |
| LT-BLEN | Vaccine | USA | 153,623 | JQ083493 |
| LARYNGOVAC USA | Vaccine | USA | 153,624 | Q083494 |
| 40798/10 | Virulent | Korea | 153,649 | MH937566 |
| 30678 | Virulent | Korea | 153,659 | MH937565 |
| 0206/14 | Virulent | Korea | 153,645 | MH937564 |
| POLUVAC ILT | Vaccine | Italy | 153,650 | KP677882 |
| 193435/07 | Virulent | Italy | 153,662 | KP677883 |
| 4787/80 | Virulent | Italy | 153,653 | KP677885 |
| 757/11 | Virulent | Italy | 153,662 | KP677884 |
| WG | Virulent | China | 153,505 | JX458823 |
| K317 | Virulent | China | 151,639 | JX458824 |
| LJS09 | Virulent | China | 151,201 | JX458822 |
| V199 | Virulent | Australia | 151,630 | JX646898 |
| SA2 | Vaccine | Australia | 151,675 | JN596962 |
| CL9 | Virulent | Australia | 151,635 | JN804827 |
| ACC78 | Virulent | Australia | 151,632 | JN804826 |
| A20 | Vaccine | Australia | 151,678 | JN596963 |
| CSW-1 | Virulent | Australia | 151,671 | JX646899 |
